# Update of the therapeutic planning of irrigation and intracanal medication in root canal treatment. A literature review 

**DOI:** 10.4317/jced.55560

**Published:** 2019-02-01

**Authors:** Ilaria Prada, Pedro Micó-Muñoz, Teresa Giner-Lluesma, Pablo Micó-Martínez, Susana Muwaquet-Rodríguez, Alberto Albero-Monteagudo

**Affiliations:** 1 Licensed Dentist at Universidad Europea de Valencia, España; 2 Endodontic and restorative dentistry Titular Professor, Universidad Europea de Valencia, España; 3 Endodontic and restorative dentistry Associate Professor, Universidad Europea de Valencia, España; 4 Licensed Dentist at Universidad Europea de Valencia. Periodontology and Osteointegration Master at Universidad de Valencia, España

## Abstract

**Background:**

The success of endodontic treatment derives from the complete elimination of microorganisms capable of causing an intraradicular or extraradicular infection. To achieve a more effective eradication of these microorganisms, endodontic instrumentation must always be implemented with abundant irrigation, which has to achieve chemical, mechanical and biological effects. The irrigators most used today are NaOCl, CHX and EDTA, released into the ducts through different techniques such as syringe, manual agitation, positive or negative apical pressure, sonic or ultrasonic activation, PIPS and PDT. The objective of this review is to update the different irrigating solutions and intracanal disinfection drugs, as well as to establish an irrigation protocol in the endodontic treatment.

**Material and Methods:**

Systematic search of scientific articles in the databases PubMed, Medline and Google Scholar, with the following keywords Endodontic, Infection, Failure, Irrigation, Retreatment and Irrigation protocol. The exclusion criteria were “case report” articles and articles with a publication date prior to 2000.

**Results:**

48 articles that met the inclusion criteria were analyzed. Comparing the different articles it can be seen that the NaOCl is the “gold standard” in terms of immediate antimicrobial efficacy, followed by the CHX that has a long-term antibacterial effect. As an intra-conductive drug it is advisable to use the combination of Ca(OH)2 with CPMC.

**Conclusions:**

The most adequate irrigation protocol consists of using 2.5% NaOCl activated with ultrasound followed by a final wash with 7% MA or 0.2% CTR combined with 2% CHX.

** Key words:**Endodontic failure, endodontic infection, enterococcus faecalis, endodontic retreatment, irrigation, sodium hipoclorite, irrigation protocol.

## Introduction

The root canal treatment success depends on a correct chemomechanical disinfection to eliminate the pulp tissue, the remains of dentin and microorganisms, thus eliminating the etiological factors that cause the endodontic infection. Therefore, the root canal instrumentation must always be accompanied by irrigation to remove the remains of pulp tissue and dentin. Without irrigation, material remains would accumulate causing the instruments to become ineffective (1).

The effects to be achieve with irrigation in endodontics are mainly three:

Chemicals: dissolution of organic and inorganic tissue, removal of dentine and smear layer residues. These effects can be expected only from chemically active irrigators (sodium hypochlorite, EDTA) (2).

Mechanics: canal lubrication, mechanical removal of microorganisms/biofilms, pulp tissue remnants, as well as the remains of dentin thanks to the forces applied by the irrigant flow. These effects can be expected both from chemically active irrigators (sodium hypochlorite) and from inert irrigants (water, saline) (2).

Biological: efficacy against anaerobic and facultative microorganisms, biofilm eradication or in activation, endotoxins inactivation (3).

The irrigating solutions classification are summarized in  Table 1 (1).

**Table 1:**
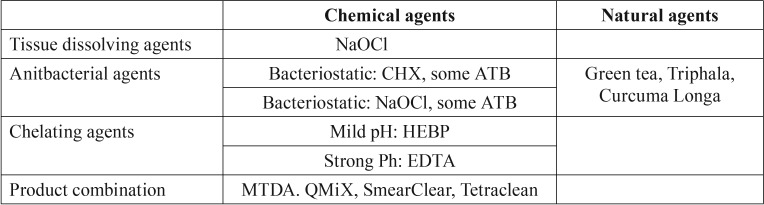
Ideal endodontic irrigation solution characteristics.

One of the biggest irrigation challenges is that it has to reach areas that the mechanical instrumentation with files does not reach, that is, the isthmus, the lateral ducts, the apical deltas, the outermost portions of the oval ducts, etc.; in fact, it is well documented that between 35% and 53% of canal wall remain uninstrumented (4). Therefore, microorganisms located in these portions have a greater survival chance (3). So the only way to eliminate the remains of tissue and microorganisms that remain in these areas is through chemical preparation with irrigants (1).

For a correct irrigation, a fundamental factor is the irrigant volume, the greater the volume, the greater the cleaning. Therefore, different and numerous methods of irrigating substances application and agitation substances have been developed (5).

The syringe release consists of transporting the solution to the canal by means of a syringe, which serves to introduce it accurately, replace the liquid, eliminate large residual particles and allow direct contact with microorganisms in the areas where the needle tip arrives. In addition, for disinfectants to effectively reach the full canal length, it is advisable to perform coronoapical movements with the irrigation needle or shaking movements with small endodontic instruments or push-pull manual movements with a gutta-percha cone (6).

Irrigation with negative pressure is used in order to improve the access of the irrigating solution access. The technique consists in applying the irrigant in the access chamber and in the root canal a very fine needle is placed which is connected to the suction device of the dental unit. Thanks to the pressure created, the excess of the irrigating solution placed in the access cavity is displaced apically and is eliminated through the suction device. This system is marketed under the name of EndoVac® (5).

Recently, in view of the need to improve the root canal disinfection, irrigation techniques have emerged whose system is based on the irrigant agitation in order to improve its diffusion and activity (6).

The sonically activated irrigation, represented mainly by the EndoActivator®, uses tips that are passively activated at 10,000 cycles / minutes for 30-60 seconds. In contrast, ultrasonic devices require vibrations greater than 20,000 Hz to give rise to the cavitation effect that allows the root canals disinfection (5).

The Photon Induced Photoacoustic Streaming (PIPS) is a new technique of laser agitation, erbium laser: garnet yttrium and alumina (ER: YAG), which has been proven effective for the debridement and the smear layer elimination, thanks to its novel design. The technique consists of placing the laser tip only in the pulp chamber without deepening to the root canal (7). This technique, to guarantee the irrigant activation, does not need the tips to move inside the canals, but it is the photo acoustic shock wave, created by the laser effect, which activates the irrigating solution and causes its three-dimensional movement in the duct system (8).

Photodynamic therapy (PDT) uses a photosensitizer (PS) that is applied in selected tissues and consists of a dye, such as malachite green, which is fixed to oral microorganisms; when the PS is exposed to a specific wavelength, low-power laser light is excited and produces a series of molecular energy transferences that result in the release of oxygen ions and free radicals, which being highly reactive and cytotoxic, they produce cell death (9).

The most known and used irrigating agents today are sodium hypochlorite (NaOCl), chlorhexidine (CHX) and ethylenediaminetetracetic acid (EDTA). None of these substances is the ideal irrigator, all have advantages and disadvantages, and because of this it is convenient to use them in combination. The market is always launching new compounds or new alternatives to enhance the effects of existing irrigants. It would therefore be interesting to compare the efficacy of the old and new irrigants on the endodontic microbiota and to see if one method is more effective than another when eradicating the bacterial biofilm, until an ideal protocol is determined.

The objectives of this bibliographical review are to compare the different therapeutic alternatives of irrigation and available intracanal drugs and to establish the most effective irrigation protocol nowadays.

## Material and Methods

The article search was carried out by one researcher in the following databases:

Pubmed: the keywords used, combined between them, Endodontic, Infection, Enterococcus faecalis, Failure, Irrigation, Retreatment, united by the Boolean AND and limiting the search field of these words in the title and in the abstract. This search gave us a total result of 1245 articles.

Medline: this search was carried through the Discover library of the European University of Valencia using these keywords: Endodontic irrigation and Retreatment, united by the Boolean AND. This search gave us a total result of 20 articles.

Google Scholar: to perform the search, the following phrase was used: Irrigation protocol in endodontics retreatment. This search gave us a total result of 2070 articles.

The inclusion criteria for the articles selection were: Articles published after 2000, “full text” articles, journal articles with an “impact factor” greater than 1, literature review articles and research articles.

“Case report” articles and articles with publication date prior to 2000 were excluded.

A total of 3335 articles have been initially located. Many of these, 1927, were duplicated in the different databases, therefore, they were eliminated, reaching a total of 1408 articles. Reading the title of each article, taking into account the objectives of the work, another 1175 records were eliminated, thus reaching a total of 233 articles. Of these, the summary was read and 135 others were eliminated that were not considered relevant for the review. Finally, 98 articles were left for the full-text review; of these, 63 articles were excluded for not complying with the inclusion parameters. To these articles were added another 13 extracted from the manual search.

The articles taken into consideration were finally 48.

## Results and Discussion

-Irrigation-disinfection materials

A successful endodontic treatment or retreatment is based on the combination of adequate instrumentation, irrigation and obturation of the canal system. Of these three phases, irrigation is the most important determinant when promoting the healing of pulp-periapical pathologies. This is so, because the irrigant can remove the remains of necrotic tissue and disinfect the canals, favoring the bacteria elimination or reduction, especially in those teeth with complex internal anatomy. To date, a large variety of irrigants has been used for this purpose, with NaOCl being the gold standard (1). In fact, the study by Giardino *et al.* (10), in 2007, carried out to evaluate the antimicrobial efficacy of 5.25% NaOCl, of Tetraclean® (a mixture of doxycycline, citric acid and detergents) and of MTAD® (a mixture of doxycycline, citric acid and detergents), confirmed the supremacy of NaOCl supremacy, since it was the only irrigator able to remove the entire biofilm after 5 min. In the same time period Tetraclean® was able to remove 90% of the biofilm, reaching 99.9% after 30 min and 100% at 60 min; whereas MTAD® was never able to completely eradicate biofilm (10). However, two years later, the same authors compared the effects of 5.25% NaOCl, Tetraclean®, Cloreximid® (a mixture of CHX and Cetrimide) and MTAD® against two different bacterial groups: bacteria strict anaerobes, represented by Prevotella and by Porphyromonas, and facultative anaerobic bacteria. In the first group, NaOCl was more effective, with statistically significant differences compared to the other irrigants, while NaOCl was not equally effective against *E. faecalis*, being overcome, with statistically significant differences, by MTAD® and Tetraclean® that led to wider inhibition zones. Cloreximid®, in both groups, was the one that showed the least antibacterial action (11).

Completely opposite are the results obtained by Dunavant *et al.* (12) in 2006 that placed the MTAD® in last position with a 16% lethality against *E. faecalis*; probably these results are due to the fact that the study by Giardino *et al.* (11) has been carried out on planktonic cells of *E. faecalis*, while the study by Dunavant *et al.* (12) was on biofilms of the same bacteria. These authors determined that the most effective antimicrobial agent is 1% and 6% NaOCl, without statistically significant differences between the two concentrations but between the same and the other irrigants analyzed: Smear Clear® (a mixture of EDTA, Cetrimide and polyoxyethylene), CHX, REDTA® and MTAD®, which achieved a case-fatality rate of 78%, 60%, 26% and 16% respectively (12). In contrast, in the study by Gomes *et al.* (13), in 2001, the three irrigating solutions that led most rapidly (<30s) to the elimination of 100% of *E. faecalis* were 5.25% NaOCl and the CHX liquid at 1% and 2%, with statistically significant differences with respect to the other concentrations of NaOCl and the CHX in gel. On the other hand, Menezes *et al.* (14), in 2004, determined that a concentration of 2.5% NaOCl is not capable of completely eliminating *E. faecalis*, being the antibacterial efficacy obtained by this irrigant statistically inferior to CHX at 2 %. However, the same two irrigants work equally well against *C. albicans* since no results were obtained with statistically significant differences (14). Completely opposite were the results obtained by Hope *et al.* (15) in 2010. In effect, they determined that 1% of concentrations has a higher lethality, with statistically significant differences, against *E. faecalis*, compared with 2% CHX and the super-oxidized water. However, CHX is significantly more effective than super-oxidized water. The effectiveness of 2% CHX is also confirmed by the study by Endo *et al.* (16), which states that an instrumentation accompanied by an irrigation with CHX eliminates 99.61% of the bacteria. A feature that makes CHX so effective against *E. faecalis* may be its ability to decrease adhesion of the bacteria adhesion to dentinal walls (17). In fact, when the CHX is used as the last irrigant, the number of bacteria that remain attached to the root canals surface is 19-28% compared to when EDTA or NaOCl are the last, respectively with 67% and 40-49% of attached bacteria (17). Because EDTA is a chelating agent that opens the dentinal tubules and exposes collagen, some authors believe that this action favors bacterial colonization (17) while other authors investigate this substance, as well as other chelating substances (phosphoric acid, citric acid), to be used as an antimicrobial agent (18). Undoubtedly, in the end, it turns out that EDTA does not possess any antimicrobial action against *E. faecalis* even after leaving it for 60 minutes; on the other hand, 2.5% phosphoric acid used 5 min to eliminate *E. faecalis* and 5% 3 min, and citric acid 25% 3 min and 10% 10 min (18). Another study that highlights the EDTA ineffectiveness is that of Baca *et al.* (19) in 2011. Comparing the antimicrobial efficacy of 17% EDTA with that of 2.5% NaOCl, of 0.2% cetrimide (CTR), of 7% maleic acid (MA) and 2% CHX, it was found that EDTA eradicates only 44% of the biofilm with statistically significant differences with respect to the other groups. The irrigator that obtained better results is 2.5% of NaOCl that only after 1 min eradicated 100% of bacteria, but without showing statistically significant differences with the CTR; however, CHX and MA eliminated 99% of the biofilm without statistically significant differences between them (19). Ferrer and Arias (20), in 2010, lowering the MA concentration to 0.88%, discovered that it is capable of completely eradicating *E. faecalis* after 30 s; the same result is obtained if 7% MA is combined with 0.2% CTR; on the other hand, if 0.2% CTR is combined with 15% citric acid or EDTA, *E. faecalis* is eradicated in 1 min, without statistically significant differences between the two combinations. These results underscore the MA ability to eliminate *E. faecalis* not only at the recommended concentration of 7% but also at a much lower concentration, 0.88%. Its antimicrobial activity may be due to its organic acid chemical nature; organic acids lower the microbial cells internal pH by altering the membrane permeability (20).

In addition to the irrigating solutions necessary to carry out a correct chemo-mechanical instrumentation, in the canals can also be introduced, especially in cases of endodontic failure, drugs such as Ca(OH)2; however, there are controversial opinions on its use and efficacy, given that microorganisms often turn out to be resistant to this disinfection measure (21). However, the study by Evans *et al.* (22), in 2002, underlines the Ca(OH)2 importance: in fact, after having exposed *E. faecalis* to Ca(OH)2 with a pH of 11.1, has been seen that only 0.4% of microorganisms survive; undoubtedly, increasing the pH to 11.5 also increases the lethality, reaching 99.9% (22). In contrast, the study by Beus *et al.* (23), in 2012, concludes that Ca(OH)2 is not strictly necessary and useful when it comes to reduce bacterial contamination of the root canals, given that the differences in the results obtained before and after the intracanal medication placementare not statistically significant, with pre-medication negative cultures being 82% and post-medication being 87%. The same results are also obtained by Endo *et al.* (16), in 2013, who concluded that there are no statistically significant differences between the samples taken after the instrumentation and after the placement of the intracanal drugs. They also found that there are no statistically significant differences between the different drugs groups analyzed, the Ca(OH)2 + CHX at 2%, which led to a decrease of the colony forming units (CFU) decrease of 99.86%, Ca(OH)2 + NaOCl at 0.9% with a decrease of 99.6% and CHX at 2% with 99.57% (16). It has been seen that CHX is capable of conferring a greater antimicrobial action when several intracanal drugs are combined. In fact, in the study of Lima and Siqueira’s study, in 2001, they saw that, among all the drug groups analyzed, only those containing CHX were capable of completely eliminating the biofilm of *E. faecalis* (24). In particular, those with greater antimicrobial efficacy with statistically significant differences with respect to the other groups were 2% CHX with 2% Natrozol, 2% o CHX with 1.25% sodium lauryl sulfate and 2% Natrozol, and 2% CHX with 15% zinc oxide, 1.25% sodium lauryl sulfate and 2% Natrozol (24). In addition to combining several drugs already known, it has been tried to introduce alternative drugs such as Tricresolformalin, canforated paramonochlorophenol (CPMC) and furacine paramonochlorophenol (FPMC). However, it has been seen that against Candida albicans Tricresol is the least effective medicine, since it leaves between 400 and 500 CFU, with statistically significant differences with respect to Ca(OH)2, CPMC, Ca(OH)2 + CPMC and FPMC. For *E. faecalis*, however, the least effective drug is FPMC with statistically significant differences with respect to Ca(OH)2 + CPMC. Therefore, a valid alternative to Ca(OH)2 alone is to combine it with CPMC, a phenolic compound that has bactericidal activity since it breaks cytoplasmic membranes, denatures proteins and inactivates enzymes (14). Also Siqueira *et al.* (25), in 2007, confirmed that the combination of Ca(OH)2 with the CPMC paste, placing it for 7 days, increases the increase negative cultures number up to 90.9% with statistically significant differences with respect to pre and post instrumentation cultures. In addition, in the same year, the same author (26) determines that the Ca(OH)2 used alone does not give statistically significant results, given that the negative cultures percentage after instrumentation is 54.5% and the post medication is 81.8. %. The ineffectiveness of Ca(OH)2 alone is also confirmed in the Siqueira and Rocas study of 2001 (27). Indeed, comparing the antifungal activity of different drugs, it turns out that the most effective are calcium sulfate combined with CPMC and Ca(OH)2 always combined with CPMC, which exceed, with statistically significant differences, Ca(OH)2 and calcium sulfate alone, which has no inhibitory properties (27). A substitute option for Ca(OH)2 may be the ozone oil hydrolysis proposed by Silveira and Siqueira in 2017 (28). Ozone owes its bactericidal, virucidal and sporicidal activity to its ionizing properties. The hydrolysis of the ozone oil can generate hydrogen peroxide, which causes the rupture of the cytoplasmatic membrane ropture, the enzymes oxidation and the damage to DNA, aldeides and ketones, which inhibit the metabolism of the bacteria metabolism and favor the rupture of the cytoplasmatic membrane ropture, thus leaving the intracellular constituents. The success rates of teeth treated with this therapy are 77%, results comparable to those obtained by the CPMC, with 74%, and with statistically significant differences with respect to those teeth that were treated in a single visit, with a success rate of 46%. Therefore, ozone oil can be a valid alternative to the common intracanal medications (28).

A very important factor that must be taken into consideration when choosing which irrigants to use during the preparation of the root canals is the substantivity, that is, the ability of the irrigating agent to continue exercising its antimicrobial action over time. This property is typical of CHX which, thanks to its cationic nature, is able to adhere to the entire canal system surfaces and remain stored there releasing slowly (1). Numerous, therefore, are the studies that test its antimicrobial efficacy in long term. One of these is that of Khademi *et al.* (29), in 2006, which compares the antimicrobial substantivity of CHX, doxycycline and NaOCl against *E. faecalis*. At day 0 the irrigant that has the highest antimicrobial activity is NaOCl, however on days 14, 21, 28, it is CHX that shows the greatest decrease in CFUs with statistically significant differences with respect to the other groups. Another study confirming that the residual effects of CHX are greater than those of NaOCl is that of Dametto *et al.* (30) in 2005. In effect, they discovered that at day 0, unlike the previous study, there are no statistically significant differences between 5.25% NaOCl and 2% CHX both liquid and gel; but on day 7 there are statistically significant differences between CHX and NaOCl, being the two CHX presentation forms equally effective in long term. In 2015, Ferrer *et al.* (31) decide to see if by lowering CHX concentration from 2% to 0.2% its antimicrobial efficacy was maintained and they also wanted to compare it with the 0.2% CTR. However, it turned out that it is 2% CHX that shows a greater inhibitory capacity at 50 days of its placement with only 34.61% growth of *E. faecalis* with statistically significant differences with respect to the other two groups. The 0.2% CHX and the CTR obtained a much higher bacterial growth of 69.23%, without statistically significant differences between them (31). Contraries are the results obtained by Baca *et al.* (19) in 2011. They discovered that 0.2% CTR is able to obtain, like CHX at 2% CHX, 100% growth inhibition of *E. faecalis* in long term, with statistically significant differences with respect to the other groups analyzed. The other irrigating solutions considered were the MA that was found to have a 85.66% bacterial inhibition, with statistically significant differences with respect to the control group, but not with respect to the results obtained by EDTA, with a 64.21% inhibition; NaOCl was also analyzed at 2.5%, which only led to a 18.10% decrease, without statistically significant differences with respect to the control group (19). Another author who wanted to analyze the long-term efficacy of 7% MA was Ferrer in 2015 (32). From this study it turned out that the best options in terms of long-term antimicrobial action, 60 days, are the combination of 7% MA with 2% CHX, with a 41.66% bacterial growth, and 0.2% CTR with 2% CHX, with a 33.33% bacterial growth, without statistically significant differences between them, but with respect to the other groups analyzed, that are: the 5.25% NaOCl, which turns out to be the worst, 100% of bacterial growth, with statistically significant difference with respect to the others, 7% MA, which, with a 91.66% growth, shows statistically significant differences with respect to CHX + MA and CHX + CTR, but not with respect to MA + CTR with a growth of 58.33% (32). Some authors maintain that NaOCl achieves such low long-term results because, due to its high surface tension, it is unable to penetrate the dentinal tubules; therefore, it would be convenient to add a detergent as a surfactant substance. This is how Hypoclean® was born, an irrigating solution based on detergents, composed of 5.25% NaOCl and two detergents (33). Two other solutions containing detergents are MTAD® and Tetraclean®. Among these three, the irrigator with the best results is Tetraclean®, with statistically significant differences with respect to NaOCl, Hypoclean® and MTAD® (33).

-Irrigation techniques

Many times these irrigating solutions are not used alone, but are usually activated with different methods so that they reach the entire canal system and, therefore, eliminate the greatest number of bacteria and organic substances (7). Numerous are the studies that compare the different activation mechanisms between them and with the conventional needle technique. Beus *et al.* (23), in 2012, compared the effectiveness of ultrasonic passive irrigation (PUI) with active ultrasonic irrigation (NUI) and found that although PUI results in 84% of negative cultures and NUI in 80%, there are no statistically significant differences between them. However, PUI can present a series of advantages: a more effective removal of pulp and dentin remnants, a greater efficiency when removing bacteria compared with manual irrigation, greater efficiency in curved canals and in cleaning the isthmuses and a great dentin removal compared to the sonic irrigation (34). It has also been shown that PUI has a very good penetration capacity in the dentinal tubules (6). The antibacterial effectiveness of the ultrasonic irrigation is also confirmed by the study by Nakamura *et al.* (35), in 2017. The authors saw that by activating the irrigating solutions with the ultrasound, in the collected samples, after the same activation, the bacteria number decrease was higher, with statistically significant differences compared to those taken after manual irrigation. However, with regard to endotoxins, no statistically significant differences were found between the two methods used. Paragliola *et al.* (6), in 2010, evaluated the NaOCl penetration when activated with different methods, and saw that the best results, with statistically significant differences, were always obtained by the PUI, in particular by the EMS® and the Satelec ®, surpassing the activation with manual files, with gutta-percha, with the EndoActivator® and with the Plastic Endo®. In contrast, PUI appears to be less effective when assessing apical safety. In fact, in the study by Desai *et al.* (5), in 2009, the EndoVac® group was the only one that did not cause apical extrusion; however, there are no statistically significant differences between this technique and the EndoActivator®, but there are differences with the ER, manual irrigation and the UI. Therefore, the placement of a microcannula at WT, typical of the EndoVac and thanks to which 50% of the irrigating substance circulates in the most apical millimeters of the root canals, is an improvement over manual irrigation, although its performance in the apical portion it is not 100% (5).

Recently, when talking about endodontic irrigation, the PDT concept has been introduced in order to minimize or eliminate residual bacteria in the root canals. Therefore, several studies compare the antimicrobial efficacy of this technique with the more conventional irrigation techniques (9), with controversial results. The study by Vaziri *et al.* (36), in 2012, confirmed that the combination of PDT with 2.5% NaOCl is capable of eliminating 100% *E. faecalis* bacteria, leading compared with a statistically significant decrease in CFUs, compared with the irrigation with PDT alone or with 2% CHX. If, on the other hand, the PDT is compared, not with manual irrigation techniques, but with activation techniques, the results are different. In fact, in the study by Xhevdet *et al.* (37), in 2014, comparing PDT with NaOCl and PUI combined with NaOCl, it is seen that the combination of PUI and NaOCl is the one that gives a better antimicrobial action against *E. faecalis* and against *C. albicans*, since it leads to a statistically greater decrease in CFU compared to the other groups analyzed.

Another innovative method, which has been introduced in the market to allow the irrigating solutions exchange fluids and the elimination of organic tissue and microorganisms, especially those with a complex internal anatomy, is the PIPS (38). Al Shahrani *et al.* (38), in 2014, demonstrated that PIPS is useful in increasing the antimicrobial efficacy of NaOCl because it was found that the largest reduction of *E. faecalis* colonies occurred in the PIPS group and 6 % NaOCl; however, the three groups analyzed, PIPS with saline solution, 6% of NaOCl and PIPS with NaOCl, led to a statistically significant decrease in CFU compared to the control group. Another study, confirming that the true antimicrobial efficacy of this method is given by 51 NaOCl effect, is that of Pedullà *et al.* (39), in 2012. Indeed, these authors demonstrated that between irrigation with NaOCl with laser activation and without laser activation there are no statistically significant differences given that both methods lead to a significant decrease in CFU compared with irrigation with distilled water with or without laser activation. However, it is the NaOCl group with PIPS activation that leads to a greater decrease in bacteria, which confirms the fact that PIPS can be used as an additive method to potentiate the effect of NaOCl (39). Another study confirming that PIPS does not provide statistically significant differences to conventional irrigation is that of Zhu *et al.* (7), in 2013. Indeed, these authors found that there are no statistically significant differences in the level of antimicrobial efficacy between CHX, NaOCl with EDTA and NaOCl with PIPS (7). Therefore, it can be concluded that PIPS can increase the NaOCl efficacy, favoring its penetration and giving it a greater bactericidal power (39).

-Clinical protocols

Due to the fact that it is not possible to determine beforehand the canal treatment, which microorganisms are present, we can not choose, with consequence, a single irrigator. That is why there is no one ideal and perfect solution for all cases, hence the importance of adopting an irrigation protocol, to achieve maximum root canal disinfection. Thus, although NaOCl possesses many qualities and properties, by itself it is not capable of totally cleaning the root canal system from organic and inorganic remains (1). Therefore, for optimal irrigation, different irrigating solutions have to be combined.

**Figure 1:**
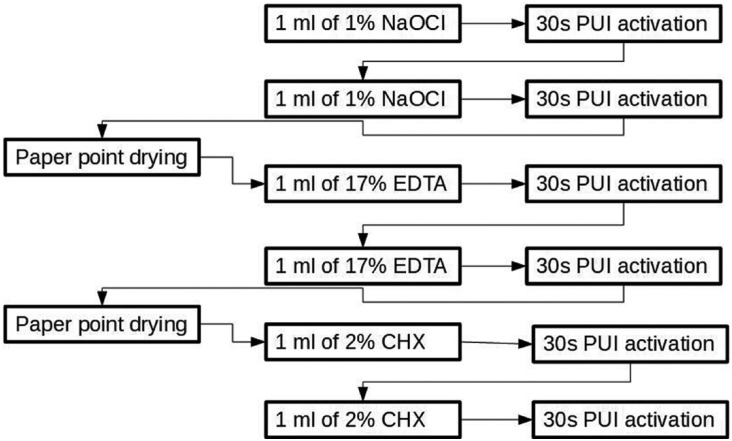
Irrigation protocol with PUI (23).

Beus *et al.* (23), in 2012, presented an action protocol combining several irrigants and choosing PUI activation method (Fig. 1). However, comparing the passive ultrasonic activation method with the non-ultrasonic activation method, which consists of pouring into the ducts 6 ml of 1% NaOCl with a continuous flow of 2 ml/min, it turns out that there are no statistically significant differences between the two protocols.

**Figure 2:**
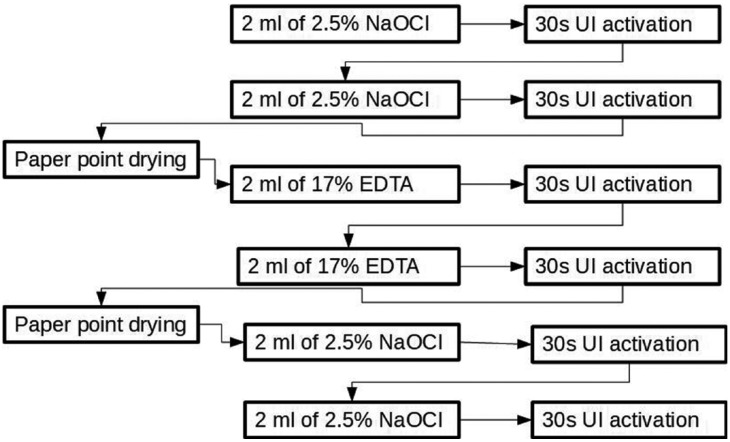
Irrigation protocol with UI (34).

On the other hand, the study by Nakamura *et al.* (35), in 2018, determines that by activating the irrigating solutions with ultrasounds, it is possible to obtain statistically significant differences following the protocol proposed in Figure 2. The differences obtained with the Beus’ study are probably due to the amount of irrigant used, in this study it is duplicated with respect to Beus’ and to the fact that in the previous study the ultrasonic irrigation was passive while in this study it is active (23) (35). The difference between the two is that in the first the tultrasound tip does not come into contact with the dentinal walls, while in the active activation the tip touches the walls and instruments simultaneously (35).

On the other hand, the results obtained by Hertel *et al.* (40), in 2016, are similar to those of Beus: applying a conventional irrigation protocol with 1% NaOCl throughout the instrumentation and a final wash with 2 ml of NaOCl during 30s there are no statistically significant differences with respect to the PUI protocol. This second protocol consists of combining 1% NaOCl with activation with PUI during the instrumentation followed by a final wash with 2 ml of 1% of NaOCl activated during 30s with PUI and with 2 ml of 20% EDTA activated during 30s with PUI. The success rate of the first protocol is 72.6% while that of the second is 82.8%. The study by Kishen *et al.* (17), in 2008, on the contrary, states that when EDTA is used as a last irrigator, this increases the number of *E. faecalis* bacteria adhered, therefore, it is advisable to irrigate, applying in sequence, as last wash, EDTA, NaOCl and CHX, given that this protocol results in the lowest number of bacteria adhered, that means 19%. Baca *et al.* (19), however, suggest that, as an irrigation protocol, to achieve the eradication of *E. faecalis*, the following is more indicated: irrigation during the instrumentation with 2.5% NaOCl, which confers an immediate antimicrobial action and a final irrigation with 2.5% NaOCl, followed by 7% MA followed by 0.2% CTR or 2% CHX, which confers 100% inhibition of bacteria in long term. Four years later, in 2015, the study by Ferrer *et al.* (32) confirms that to effectively and in the long term eliminate *E. faecalis* it is convenient to use for the final irrigation 7% MA or 0.2% CTR; the only difference marked with the previous study is that Ferrer and cols. advise to use them always combined with the 2% CHX since the result obtained by this combination shows statistically significant differences with respect to the agents used alone (32).

## Conclusions

The “gold standard” irrigant in terms of immediate antimicrobial efficacy, with statistically significant differences, remains the NaOCl, but without obtaining unanimity on the ideal concentration to be used, which ranges between 0.5% and 6%. In second position CHX at 2%, is placed which nevertheless exceeds, with statistically significant differences, NaOCl and all other solutions available in the market in terms of long-term efficiency. Regarding intracanal medications, there are controversies about the use of Ca(OH)2 alone; the combination of Ca(OH)2 with CPMC seems promising. The activation method that has been shown to be most effective is ultrasonic activation. PIPS and PDT also lead to a significant decrease in the number of bacteria. However, all these agitation methods are practically comparable with manual irrigation.

The most effective irrigation protocol to eliminate *E. faecalis* responsible for the majority of endodontic failures consists of: I) Irrigation with 2.5% NaOCl, II) Choice of LAM, III) irrigants activation with ultrasound by the following form: 2 ml of 2.5% NaOCl plus 30s of activation with UI (x2); aspirate NaOCl; 2 ml of 17% EDTA plus 30s of activation with UI (x 2); aspire EDTA; 2 ml of 2.5% NaOCl plus 30s of activation with UI (x 2), IV) Final wash with 7% MA + 2% CHX or 7% MA + 0.2% CTR + 2% CHX.
